# Effective Botulinum Toxin Injection Guide for Treatment of Temporal Headache

**DOI:** 10.3390/toxins8090265

**Published:** 2016-09-08

**Authors:** You-Jin Choi, Won-Jae Lee, Hyung-Jin Lee, Kang-Woo Lee, Hee-Jin Kim, Kyung-Seok Hu

**Affiliations:** Division in Anatomy and Developmental Biology, Department of Oral Biology, Human Identification Research Institute, BK21 PLUS Project, Yonsei University College of Dentistry, Seoul 03722, South Korea; cyj7797@yuhs.ac (Y.-J.C.); enamelee@daum.net (W.-J.L.); leehj221@yuhs.ac (H.-J.L.); leekw@yuhs.ac (K.-W.L.); hjk776@yuhs.ac (H.-J.K.)

**Keywords:** migraine, botulinum toxin type A, temporalis muscle, injection site, Sihler staining

## Abstract

This study involved an extensive analysis of published research on the morphology of the temporalis muscle in order to provide an anatomical guideline on how to distinguish the temporalis muscle and temporalis tendon by observing the surface of the patient’s face. Twenty-one hemifaces of cadavers were used in this study. The temporalis muscles were dissected clearly for morphological analysis between the temporalis muscle and tendon. The posterior border of the temporalis tendon was classified into three types: in Type I the posterior border of the temporalis tendon is located in front of reference line L2 (4.8%, 1/21), in Type II it is located between reference lines L2 and L3 (85.7%, 18/21), and in Type III it is located between reference lines L3 and L4 (9.5%, 2/21). The vertical distances between the horizontal line passing through the jugale (LH) and the temporalis tendon along each of reference lines L0, L1, L2, L3, and L4 were 29.7 ± 6.8 mm, 45.0 ± 8.8 mm, 37.7 ± 11.1 mm, 42.5 ± 7.5 mm, and 32.1 ± 0.4 mm, respectively. BoNT-A should be injected into the temporalis muscle at least 45 mm vertically above the zygomatic arch. This will ensure that the muscle region is targeted and so produce the greatest clinical effect with the minimum concentration of BoNT-A.

## 1. Introduction

Headaches can be clinically classified into primary and secondary types. Primary headaches occur without underlying organic diseases and can be further classified into migraine, tension-type headaches, and cluster headaches. Among the three classifications, migraines have the strongest hereditary associations. Patients suffering from migraines often show hypersensitive reactions to light, sounds, and touch, and during severe episodes this can lead to photophobia, acousticophobia, and olfactophobia. Although migraines are not life-threatening, they can severely impair the everyday lives of affected patients.

The underlying causes of migraines are often nerve and muscle disorders, which has led to botulinum toxin type A (BoNT-A) gaining traction as viable treatment optio [1,2,3] BoNT-A reportedly has fewer side effects than the drugs that are commonly used for headache. Furthermore, a single BoNT-A treatment can last up to 4 months, in contrast to the fairly short-term effects of orally administered drugs and lidocaine injections [4,5]. The US Food and Drug Administration (FDA) recognized the safety and effectiveness of BoNT-A by approving 31 injection sites for BoNT-A on October 2010. Among them, four injection sites on each side of the temporalis muscle were suggested. These injection sites were approved after observing the clinical ramifications of BoNT-A injections originally aimed at treating tension-type headaches. However, anatomical research focused on the neural distribution of the head and neck area is needed to form a basis for accurate and specific BoNT-A injections.

The specific mechanism by which BoNT-A relieves pain has not yet been identified [6,7], but it can be hypothesized that the relief of muscular tension, transcytosis and the retrograde transport of BoNT-A suppress the diffusion of neurotransmitters across the peripheral nerve [8]. These mechanisms suppress both peripheral and central sensitization, providing relief for patients suffering from peripheral and central neuritis. Since BoNT-A acts on nerve endings, an extensive and accurate anatomical understanding of the nerve endings of the targeted muscle is critical for obtaining maximum relief with the minimum concentration of BoNT-A.

Invasive anatomical procedures are of limited use when attempting to find effective BoNT-A injection sites due to the risk of damaging the muscle and the target nerve endings. In contrast, Sihler’s staining, which dyes myelin sheaths, provides a minimally invasive and effective method of tracking nerve endings within a targeted muscle [9,10]. The application of Sihler’s staining to the temporalis muscle will facilitate accurate and extensive observations of the neural distribution and nerve endings of the deep temporal nerve.

The morphology of the temporalis muscle restricts the ability to identify effective injection sites by simply observing the associated neural distribution, and the pharmaceutical application of BoNT-A in the tendon region is highly ineffective. Sihler’s staining must therefore be accompanied by morphological investigations of the temporalis muscle and temporalis tendon.

There are various techniques for treating headaches via BoNT-A injections. However, botulinum toxin injections are based on two main strategies for migraines: the pain strategy and the fixed point strategy. Though no conclusions have been reached on which method is more effective, it is important to note that both techniques require BoNT-A injections which avoid the tendon of the temporalis.

This study performed an extensive analysis of published research on the morphology of the temporalis muscle in order to provide an anatomical guideline on how to distinguish the temporalis muscle and temporalis tendon by observing the surface of the patient’s face. Furthermore, it was found that Sihler’s staining could be applied to the temporalis muscle in order to identify accurate and effective BoNT-A injection sites for treating temporal headache.

## 2. Results

### 2.1. Posterior Border of the Temporalis Tendon

**Figure 1:**
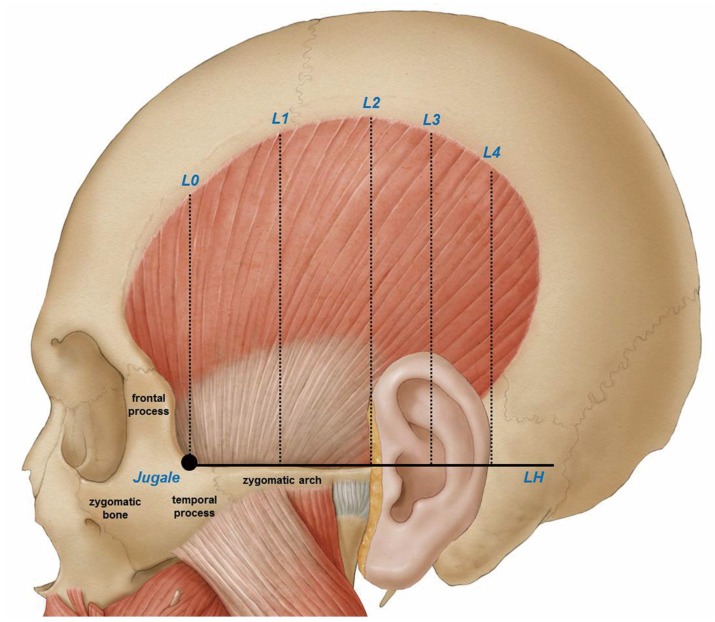
Reference points and lines based on surface anatomical structures. Jugale: A landmark on the skull at which the temporal and frontal processes of the zygomatic bone meet. LH: The horizontal line passing through the jugale. L0: The vertical line passing through the jugale. L1: The line evenly dividing L0 and L2. L2: The vertical line passing through the anterior outer margin of the ear. L3: The line evenly dividing L2 and L4. L4: The vertical line passing through the posterior outer margin of the ear.

**Figure 2:**
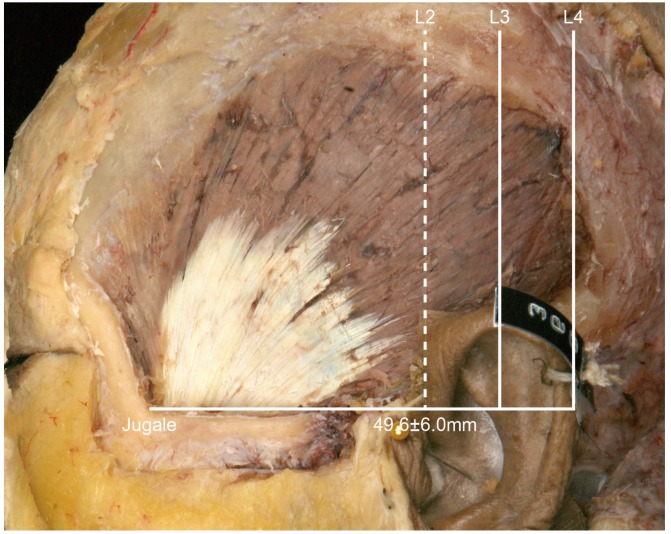
Type I, in which the posterior border of the temporalis tendon is located in front of L2 (4.8%, 1/21). The distance between the jugale and L2 was 49.6 ± 6.0 mm.

**Figure 3:**
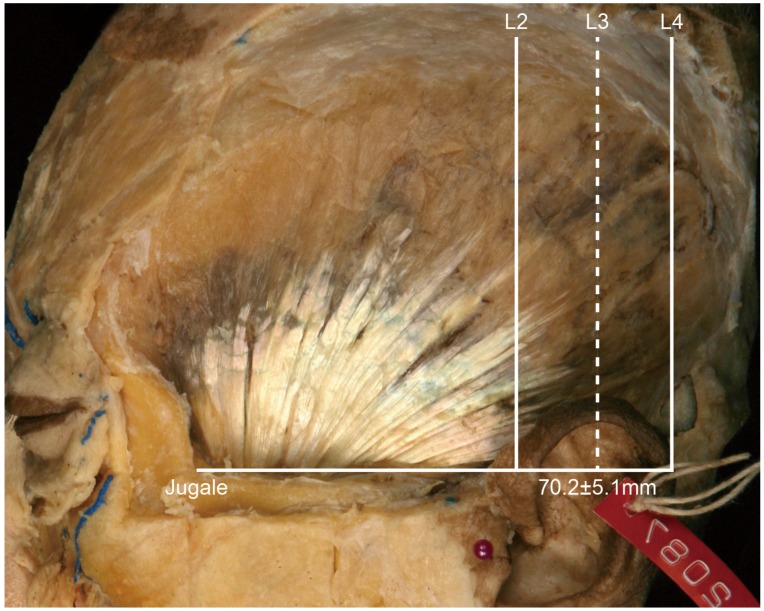
Type II, in which the posterior border of the temporalis tendon is located between L2 and L3 (85.7%, 18/21). The distance between the jugale and L3 was 70.2 ± 5.1 mm.

**Figure 4:**
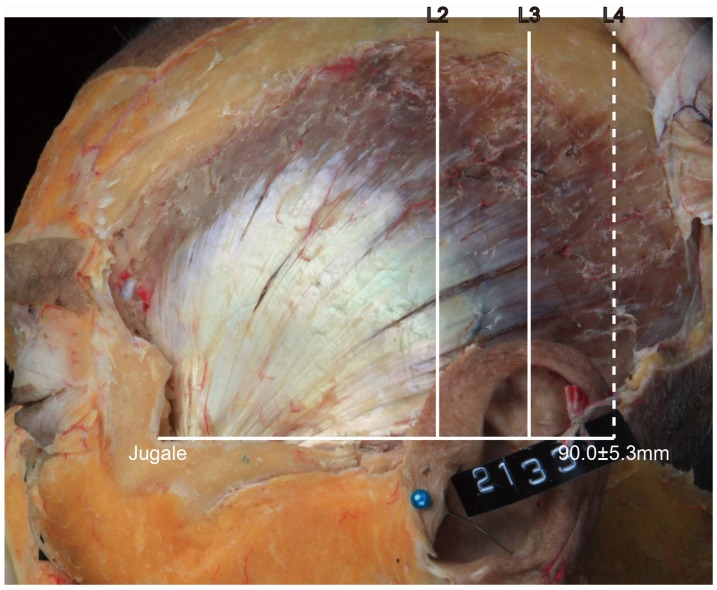
Type III, in which the posterior border of the temporalis tendon is located between L3 and L4 (9.5%, 2/21). The distance between the jugale and L4 was 90.0 ± 5.3 mm.

The ear and reference lines L2, L3, and L4 was used to clearly delineate the temporalis tendon. Details on reference line used in this study are on Figure 1. The posterior border where the temporalis tendon disperses can be classified into the following three types:

• In Type I, the posterior border of the temporalis tendon is located in front of L2 (Figure 2). In this type the distance between the jugale and L2 was 49.6 ± 6.0 mm (mean ± SD), and it occurred in 1 of the 21 cases (4.8%).

• In Type II, the posterior border of the tendon is located between L2 and L3 (Figure 3). In this type, the distance between the jugale and L3 was 70.2 ± 5.1 mm, and it occurred in 18 of the 21 cases (85.7%).

• In Type III, the posterior border of the tendon is located between L3 and L4 (Figure 4). In this type, the distance between the jugale and L4 was 90.0 ± 5.3 mm, and it occurred in 2 of the 21 cases (9.5%).

There were no cases where the tendon dispersed beyond L4.

### 2.2. Vertical Border of the Temporalis Tendon and Temporalis Muscle

The vertical distances between the horizontal line passing through the jugale (LH) and the temporalis tendon along the L0, L1, L2, L3, and L4 reference lines were 29.74 ± 6.87 mm, 45.06 ± 8.84 mm, 37.76 ± 11.18 mm, 42.50 ± 7.59 mm, and 32.14 ± 0.47 mm, respectively; the corresponding vertical distances between LH and the temporalis muscle were 55.02 ± 8.25 mm, 74.99 ± 9.90 mm, 73.97 ± 10.12 mm, 55.24 ± 13.25 mm, and 47.56 ± 11.41 mm (Table 1).

### 2.3. Intramuscular Nerve Distribution of the Temporalis Muscle

**Figure 5:**
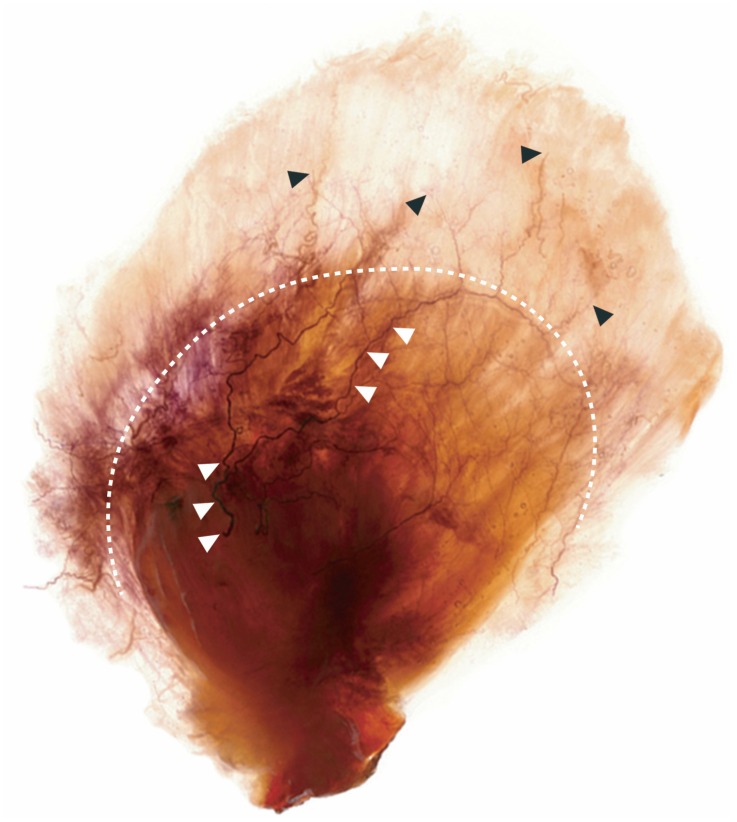
The temporalis muscle and tendon could be clearly distinguished after Sihler’s staining (white dotted line). The temporalis tendon appeared as a fan beginning at the point where it is inserted into the temporalis muscle. The temporalis muscle occupied the remaining area. The nerve trunk of the deep temporal nerve traversed the temporalis tendon (white arrows). The nerve endings of the deep temporal nerve mainly dispersed in the temporalis muscle (black arrows).

The temporalis is a masticatory muscle that can be divided into anterior, middle, and posterior regions. Sihler’s staining showed that the anterior branch of the deep temporal nerve runs through the anterior fibers of the temporalis muscle, which provides upward elevation of the mandible. The posterior branch of the deep temporal nerve runs through the posterior fibers of the temporalis muscle, which provides backward elevation of the mandible. The middle branch of the deep temporal nerve runs through the middle fibers of the temporalis muscle (Figure 5).

## 3. Discussion

Migraine treatments can be divided largely into pharmacotherapy and physiotherapy. Pharmacotherapy can be further classified into the oral administration of amitriptyline and topiramate or the injection of BoNT-A and lidocaine. Physiotherapy involves exercise, positional release therapy, and massage therapy [11,12,13]. However, the oral administration of drugs for extended periods poses a risk of significant side effects for those with cardiovascular disease, cerebrovascular disease, peripheral vascular disease, liver disease, and pregnancy. Furthermore, there is currently insufficient scientific evidence for the physiological benefits of physiotherapy [14,15].

The mechanism by which BoNT-A relieves migraines is not yet clear. There is empirical evidence suggesting that BoNT-A has minimal side effects, and a single treatment lasts up to 4 months. The FDA has approved 31 sites at which BoNT-A can be injected for treating migraines. However, since these sites were chosen based on observations of the trigger point of migraines, it is unclear whether these are the most anatomically effective sites for injection-based treatments. In order to find the most anatomically effective injection sites, an extensive study of the nerve endings in the temporalis muscle is necessary.

Sihler’s staining was used in this study to observe the neural distribution in the temporalis region. The tendon and muscle regions could be clearly distinguished after applying Sihler’s staining, which was bolder in the tendon region. The anterior, middle, and posterior branches of the deep temporal nerve traverse the temporalis tendon into the intramuscular layer. A notable characteristic of the deep temporal nerve was that all of its branches traverse without dispersion in the temporalis tendon—only when the nerve branches reached the muscle region did the nerve endings disperse.

There is anatomical evidence supporting the clinical practice of injecting BoNT-A into the temporalis muscle while avoiding the temporalis tendon, including because (1) there is pharmaceutical evidence that BoNT-A acts in the muscle region [16] and (2) the neural distribution of the temporalis muscle indicates that its nerve endings are densely located in the muscle region. This anatomical evidence can be used to produce a guideline for injection sites on the temporalis muscle. Since the temporalis region can be morphologically divided into the tendon and muscle regions, a guideline on the anatomical surfaces in which BoNT-A should be injected can be suggested. Based on the results of this study, we suggest that the temporalis tendon is horizontally located approximately 45 mm from LH, and any location more than 45 mm vertically above LH can be considered the muscle region.

The jugale is a part of the zygomatic bone that horizontally follows the superior margin of the zygomatic arch. The horizontal line LH, which passes the jugale, coincides with the superior margin of the zygomatic arch. It can therefore be clinically applied as the superior margin of the zygomatic arch. We conclude that the temporalis tendon stretches 45 mm from the superior margin of the zygomatic arch.

**Figure 6:**
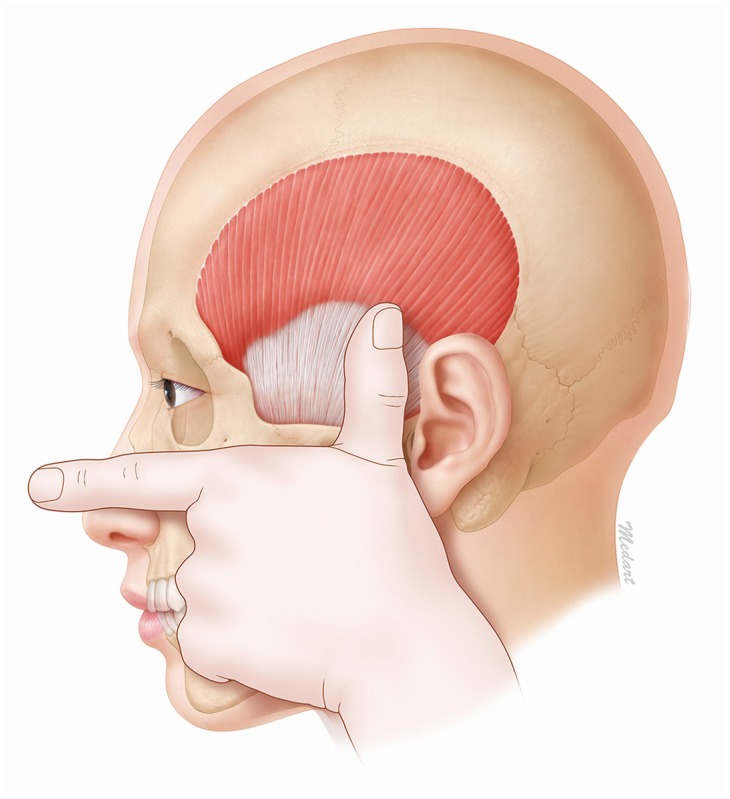
The position of the temporalis tendon can be easily determined first aligning the thumb and first finger in a flat stretched-out state and then placing the second finger on the inferior margin of the zygomatic arch. The tip of the thumb is then located approximately 45 mm from the superior margin of the zygomatic arch. However, since every clinician varies in thumb length, we recommend that a clinician use the above-mentioned method after taking into consideration the difference when comparing the point 45 mm from the patient’s zygomatic arch with that of the location of his/her own thumb.

The temporalis tendon can be easily found by first aligning the thumb and the first finger in a flat stretched-out state and then placing the second finger on the inferior margin of the zygomatic arch. This will result in the tip of the thumb being located approximately 45 mm from the superior margin of the zygomatic arch (Figure 6). This method makes it easy to identify the temporalis muscle and therefore also the effective injection site for BoNT-A.

## 4. Conclusions

An easily identifiable and effective injection site for BoNT-A on the temporalis muscle has been suggested based on the results of this research. In treatment of chronic migraine with botulinum toxins, for optimal effect, any mid-temporal injection need to be given at least 45 mm above the zygomatic arch to avoid injecting into the tendon. This will ensure that the target is the muscle region, which will produce the greatest clinical effect using the minimum concentration of BoNT-A. The main findings of this study can be summarized as follows:

- The temporalis tendon is shaped like a fan. The most distant point of the tendon region is located 45 mm from the zygomatic arch. Therefore, the site of BoNT-A injection into the temporalis muscle should be at least 45 mm from the zygomatic arch.
- Sihler’s staining shows that the nerve endings that BoNT-A acts on are densely dispersed in the temporalis muscle, which indicates that the muscle region is an effective site for BoNT-A injection.
- In order to easily identify the temporalis muscle in a clinical setting, the second finger should be placed on the bottom corner of the zygomatic arch. The tip of the thumb will then be located 45 mm from the zygomatic arch.

Clinicians with a comprehensive understanding of the results reported in this paper will be easily able to determine an effective BoNT-A injection site for migraine treatment.

## 5. Materials and Methods

### 5.1. Materials

Twenty-one hemifaces of cadavers (16 males, 5 females; mean age, 81.0 years; age range, 63–93 years) were used in this study. All of the cadavers were donated by Yonsei Medical Center. This project approved by Yonsei University Dental Hospital Institutional review board (IRB) since 15 October 2015 (IRB No. 2-2015-0039). None of the cadavers had any damage or previous surgery in the temporal region. The experiment was divided into two steps: (1) morphologically analyzing the temporalis region of the cadavers and (2) applying Sihler’s staining to the temporalis muscle and tendon.

### 5.2. Methods

#### 5.2.1. Morphological Analysis of the Temporalis Muscle and Tendon

The skin, subcutaneous tissue, and superficial and deep temporal fasciae of the temporalis muscle were first removed, which allowed the temporalis tendon and temporalis muscle to be clearly identified. This study used several anatomical points as standard markers to measure the width, height, and length of the temporalis muscle.

In order to provide a clear clinical guideline for BoNT-A injection in the temporalis region, the jugale and zygomatic arch were used as structural anatomical markers to morphologically delineate the temporalis muscle. The reference points and lines shown in Figure 1 were established prior to making the measurements described above.

Horizontal line LH was used as a standard for measuring the vertical distances separately to the temporalis muscle and temporalis tendon using vertical lines L0–L4 as defined in Figure 1. All measurements were made using a protractor and digital calipers (CD-15CP, Mitutoyo, Kawasaki, Japan) capable of measuring to the nearest 0.01 mm.

#### 5.2.2. Sihler’s Staining for Identifying the Neural Distribution of the Temporalis Muscle

Sihler’s staining was applied to reveal the neural distribution of the temporalis muscle. Sihler’s staining involves the seven steps described below. Although the duration of staining varies according to thickness, it usually takes up to 5 months.

##### Fixation

The temporalis muscle that had been dissected from the cadaver was placed in 10% unneutralized formalin (DUKSAN, Ansan, Korea) for 1 month. The duration of formalin fixation varied according to the specimen size and thickness. The formalin did need not to be replaced unless it became polluted.

##### Maceration

The fixed specimens were washed for 1 h in running water, and then stored in 3% potassium hydroxide (JUNSEI, Tokyo, Japan) solution. 3% aqueous potassium hydroxide solution containing 0.2 mL of 3% hydrogen peroxide (DUKSAN, Ansan, Korea) per 100 mL. The fixed specimens became partially transparent after 3 to 4 weeks of storage, during which time the potassium hydroxide solution was replaced daily.

##### Decalcification

The macerated specimens were stored for 4 weeks in Sihler’s solution I, which comprises glacial acetic acid (DUKSAN, Ansan, Korea), glycerin (DUKSAN, Ansan, Korea), and 1% aqueous chloral hydrate (Wako, Osaka, Japan) mixed in a 1:1:6 ratio. This solution was replaced weekly.

##### Staining

Adequately decalcified specimens were stained for 3 to 4 weeks in Sihler’s solution II, which comprises Ehrlich’s hematoxylin (Acros, Morris Plains, NJ, USA), glycerin, and 1% aqueous chloral hydrate mixed in a 1:1:6 ratio. The staining period was varied according to the specimen size and thickness. A viewing box was used to decide when the Ehrlich’s hematoxylin had permeated the specimen sufficiently.

##### Destaining

Stained specimens are again placed in Sihler’s solution I so that they became transparent. This step was necessary since Sihler’s solution II dyes both the nerves and muscle tissue purple. The specimens were checked frequently until adequate transparency was achieved. Depending on the specimen, the process took up to 2 h.

##### Neutralization

The acidified specimen was neutralized in a 0.05% lithium carbonate solution (DUKSAN, Ansan, Korea) for about 1 h before being washed in running water for 30 min.

##### Clearing

The specimen was placed in a thymol crystal containing glycerin solution. The concentration of glycerin was increased in four stages (40%, 60%, 80%, and 100% glycerin), before being finally stored in a 100% glycerin solution.

## Figures and Tables

**Table 1:** The vertical distance of the temporalis tendon and muscle from LH.

Points	The Vertical Length of the Tendon	The Vertical Length of the Muscle
L0	29.7 ± 6.8	55.0 ± 8.3
L1	45.1 ± 8.8	75.0 ± 10.0
L2	37.8 ± 11.2	74.0 ± 10.1
L3	42.5 ± 7.6	55.2 ± 13.3
L4	32.1 ± 0.5	47.6 ± 11.4

Values are expressed in millimeters (mm).
